# Perspectives of Rehabilitation Professionals on Implementing a Validated Home Telerehabilitation Intervention for Older Adults in Geriatric Rehabilitation: Multisite Focus Group Study

**DOI:** 10.2196/44498

**Published:** 2023-07-18

**Authors:** Margriet Pol, Amarzish Qadeer, Margo van Hartingsveldt, Mohamed-Amine Choukou

**Affiliations:** 1 Amsterdam University of Applied Sciences, Research Group Occupational Therapy - Participation and Environment, Faculty of Health, Center of Expertise Urban Vitality Amsterdam Netherlands; 2 Amsterdam University Medical center, location Vrije Universiteit Amsterdam, Department of Medicine for Older People Amsterdam Netherlands; 3 Amsterdam Public Health, Aging & Later Life Amsterdam Netherlands; 4 Bimedical Engineering graduate program, University of Manitoba Winnipeg, MB Canada; 5 Department of Occupational Therapy College of Rehabilitation Sciences, Rady Faculty of Health Sciences University of Manitoba Winnipeg, MB Canada; 6 Centre on Aging, University of Manitoba Winnipeg, MB Canada

**Keywords:** aging in place, aging well, digital technology, remote monitoring, activity, sensor, mobile phone

## Abstract

**Background:**

Owing to demographic trends and increasing health care costs, quick discharge with geriatric rehabilitation at home is advised and recommended for older adults. Telerehabilitation has been identiﬁed as a promising tool to support rehabilitation at home. However, there is insufficient knowledge about how to implement a validated home telerehabilitation system in other contexts. One of the major challenges for rehabilitation professionals is transitioning to a blended work process in which human coaching is supplemented via digital care.

**Objective:**

The study aimed to gain an in-depth understanding of the factors that influence the implementation of an evidence-based sensor monitoring intervention (SMI) for older adults by analyzing the perspectives of rehabilitation professionals working in 2 different health ecosystems and mapping SMI barriers and facilitators.

**Methods:**

We adopted a qualitative study design to conduct 2 focus groups, 1 in person in the Netherlands during winter of 2017 and 1 on the web via Zoom (Zoom Video Communications; owing to the COVID-19 pandemic) in Canada during winter of 2022, to explore rehabilitation providers’ perspectives about implementing SMI. Qualitative data obtained were analyzed using thematic analysis. Participants were a group of rehabilitation professionals in the Netherlands who have previously worked with the SMI and a group of rehabilitation professionals in the province of Manitoba (Canada) who have not previously worked with the SMI but who were introduced to the intervention through a 30-minute web-based presentation before the focus group.

**Results:**

The participants expressed different characteristics of the telerehabilitation intervention that contributed to making the intervention successful for at-home rehabilitation: focus on future participation goals, technology support provides the rehabilitation professionals with objective and additional insight into the daily functioning of the older adults at home, SMI can be used as a goal-setting tool, and SMI deepens their contact with older adults. The analysis showed facilitators of and barriers to the implementation of the telerehabilitation intervention. These included personal or client-related, therapist-related, and technology-related aspects.

**Conclusions:**

Rehabilitation professionals believed that telerehabilitation could be suitable for monitoring and supporting older adults’ rehabilitation at home. To better guide the implementation of telerehabilitation in the daily practice of rehabilitation professionals, the following steps are needed: ensuring that technology is feasible for communities with limited digital health literacy and cognitive impairments, developing instruction tools and guidelines, and training and coaching of rehabilitation professionals.

## Introduction

The worldwide aging revolution has put the rehabilitation of older adults high on the agenda of both health care policy and research [1]. The 2 critical policies in many resource-limited countries, aging and reducing hospitalization, which particularly affect older people who are frail, have stimulated the search for appropriate and cost-effective use of rehabilitation resources. It is crucial to increase the adoption of digital health care technologies to support the stakeholders (e, rehabilitation professionals and older adults and their families) in care pathways [2,3].

Geriatric rehabilitation (GR) is defined as “a multidimensional approach of diagnostic and therapeutic interventions, the purpose of which is to optimize functional capacity, promote activity and preserve functional reserve and social participation in older people with disabling impairments*”* [4]. GR consists of multidisciplinary care with a focus on function and participation after acute illness or functional decline [5,6]. In GR, people over the age of 75 years living with multiple comorbidities are often categorized into four groups: people with (1) stroke (21% of people); (2) traumatic orthopedic problems (19% of people); (3) elective orthopedic surgery (14% of people); and (4) other conditions (38% of people), for example, cardiac, neurological, or oncological problems [5,7]. Depending on national policies and local availability, GR may be offered community service, hospital service, skilled nursing facility, or intensive day program. This results in different patient journeys. The aim of GR is to return home. Once it is safe, based on the condition of the person and social environment, people are encouraged to be discharged home [8,9]. This decision does not mean that these older adults are fully rehabilitated and have reached their rehabilitation potential. They are often restricted in daily functioning and still dependent on ongoing support by rehabilitation professionals and informal care [10-12].

Owing to demographic trends, quick discharge with GR at home is advised and recommended. Moreover, rehabilitation at home is more realistic, and older adults report high satisfaction levels [13]. Therefore, optimal rehabilitation care beyond discharge is crucial, with particular attention to the everyday activities that are meaningful for individuals [12,14]. However, the smooth transition from inpatient GR to home is challenging [15]. The first challenge is that only a minority of older adults receive home-care rehabilitation services after discharge [12]. Second, the therapist providing in-home rehabilitation is rarely the same therapist at the institution from which the person received initial care, which undermines the continuity of the rehabilitation process. Third, working in the community differs from working in an inpatient setting and requires other skills and work routines. Being discharged from inpatient GR to home with a rehabilitation plan but without continuous support negatively influences the rehabilitation process. The lack of support has, for example, negative consequences for adherence to prescribed exercise routines [16] and leads to a sense of insecurity in older adults [12]. A fourth challenge is the lack of involvement of the older adult in decision-making related to home rehabilitation [17].

**Figure 1:**
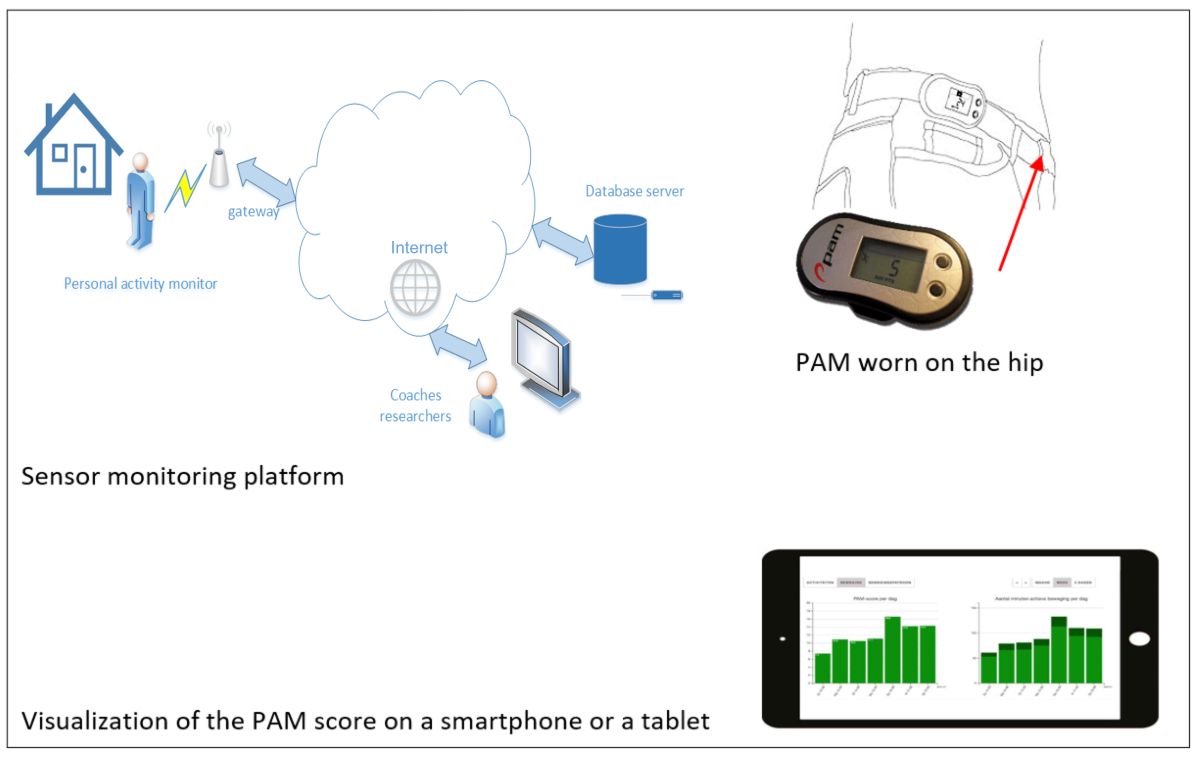
The sensor monitoring platform. PAM: physical activity monitor.

In this context, telerehabilitation has been identiﬁed as a promising tool in GR [2]. Previous studies investigating telerehabilitation in different conditions have yielded encouraging results [16,18]. A promising and effective home telerehabilitation intervention is a sensor monitoring intervention (SMI) for older individuals rehabilitating after hip fracture, developed at the University of Amsterdam and the Amsterdam University of Applied Sciences [19-21]. The intervention consists of a rehabilitation protocol of coaching supported by sensor monitoring. The coaching is based on the principles of a cognitive behavioral therapy program concerning falls and focuses on setting realistic goals for increasing performance in meaningful daily functioning at home. The sensor technology consists of a wearable sensor worn on the hip that was used to assist older adults in obtaining feedback about their daily physical functioning and as a tool to assist therapists in coaching [22,23]. The wearable activity monitor (physical activity monitor [PAM]; [24]) comprises a 3D accelerometer worn on the hip (68 × 33 × 10 mm). The sensor measures the activity level per day, expressed as a PAM score, which is the ratio between the amount of energy used while active and the amount of energy used while at rest, multiplied by 100. Furthermore, the sensor gives the number of minutes of daily regular and vigorous activity [25]. The data collected by the PAM sensor are stored in the PAM itself and are synchronized with the gateway using Bluetooth when the client is near the gateway. The PAM sensor can collect data for 64 days without synchronization and runs on a single battery for 7 months [23]. This transitional rehabilitation starts within the geriatric care facility with a follow-up rehabilitation at home. Figure 1 shows the sensor monitoring platform’s components and system interactions diagram.

The results from a randomized controlled trial (RCT) were positive. In an RCT including 240 community-dwelling older adults after hip fracture, older adults in the sensor monitoring group perceived greater improvements in daily functioning than those in the care-as-usual group [22]. Although the findings from the RCT for the SMI are positive for older adults after hip fracture, there is insufficient knowledge about how to implement a validated home telerehabilitation system in other contexts. One of the major challenges for rehabilitation professionals is the transition to a blended work process in which human coaching is supplemented by digital care. A systematic implementation approach will be crucial to understand its fit within current transitional rehabilitation from different stakeholder perspectives [26]. Therefore, the purpose of this study was to depict the factors that influence the implementation of an evidence-based home telerehabilitation intervention for older adults from the perspectives of rehabilitation professionals working in two different health ecosystems—(1) rehabilitation professionals in the Netherlands who have previously worked with the SMI and (2) rehabilitation professionals in the province of Manitoba (Canada) who have not previously worked with the SMI—by mapping of the barriers to and facilitators of using the intervention. For the sake of clarity for international readers, the term “Canada” will refer to the province of Manitoba in this paper. Our study attempts to answer the following research questions:

1. From the rehabilitation professionals’ perspectives in both contexts (the Netherlands and Canada), what are the characteristics of a successful telerehabilitation intervention in the transition from inpatient to home rehabilitation?
2. What are the needs and expected roles of technology-enabled solutions in GR at home in Canada, and what is the Dutch experience?
3. To what extent are Canada and the Netherlands’ health ecosystems ready to adopt the SMI?
4. From the rehabilitation professionals’ perspectives in both contexts, what are the barriers to and facilitators of using SMI at home?
5. What are the possible next steps to implement SMI in other contexts in Canada and the Netherlands?

## Methods

### Design

For this exploratory study, we conducted 2 focus groups (FGs), 1 in person in the Netherlands (winter of 2017) and 1 on the web via Zoom (Zoom Video Communications; owing to the COVID-19 pandemic) in Canada (winter of 2022) to explore rehabilitation professionals’ perspectives about implementing SMI. This qualitative research approach allows to gain an in‐depth understanding of the barriers to and facilitators of using SMI in the Netherlands (FG 1) or introducing SMI in the Canadian context (FG 2) [27]. The COREQ (Consolidated Criteria for Reporting Qualitative Research) checklist for reporting qualitative research was followed [28].

### Context

This study was part of an ongoing study of the development, effectiveness, and implementation of an SMI following the Medical Research Council framework, a framework for the development and evaluation of complex interventions [29]. The first phases of the framework (the development of the intervention, feasibility, and evaluation) were conducted earlier [30]. This study focused on the stage of implementation and was built on the knowledge gained from the RCT and process evaluation that we conducted alongside the RCT. There was a worldwide surge in the use of telerehabilitation technologies during the COVID-19 pandemic. Therefore, our study secondarily explored the effect of the pandemic on the rehabilitation professionals’ perspectives about telerehabilitation before and during the pandemic and using technology to support remote care.

We conducted this study in 2 international contexts. FG 1 was conducted in the Netherlands at the Amsterdam University of Applied Sciences, located in Amsterdam. Participants in this FG worked at 6 different health care organizations for GR in the Netherlands’ middle and northwest regions. FG 2 was conducted in Canada at the University of Manitoba located in Winnipeg, Manitoba. All participants in this FG work at a public rehabilitation and long-term care facility.

### Participants

#### Focus Group 1

We purposefully sampled occupational therapists (OTs) who delivered the intervention in the RCT (n=34) [22]. Participants were approached by the main researcher via a recruitment mail including an information letter.

#### Focus Group 2

Participants were approached via a recruitment email sent by the Deer Lodge Centre Foundation to the Deer Lodge Centre clinical staff. A research team member then contacted the individuals interested in participating in the study. Eligibility criteria for this FG were being an OT or physical therapist (PT) with experience in GR.

### Ethics Approval

FG 1 was approved by the Medical Ethics Committee of the Amsterdam University Medical Center located in the Netherlands (ID AMC 2015_169). FG 2 was approved by the University of Manitoba Human Research Ethics Board (HS24220 [H2020:390]).

### FG Sessions

The FG sessions followed the guidelines as described by Kruger et al [31].

FG 1 was moderated by an experienced independent moderator and coauthor, MP. The FG lasted 90 minutes. We developed and tested a topic guide to explore therapists’ experiences and opinions regarding the use of the telerehabilitation intervention (Multimedia Appendix 1).

First, participants were asked to introduce themselves and share their years of experience, where they currently work, and what type of older adults they deal with. Second, the FG discussion goals were shared with all the participants. Third, brainstorming with stick notes was conducted to collect the most important topics, and questions and discussions were followed according to the topic guide.

FG 2 was moderated by 2 coauthors (MAC and AQ), was conducted via Zoom, and lasted 1 hour. The researchers followed an FG guide (Multimedia Appendix 1). The discussion started with a general introduction of MAC and AQ. Participants were also asked to introduce themselves and share their years of experience, where they currently work, what type of older adults they deal with, and the focus of their work. Then, the purpose of the FG discussion was shared with all the participants, followed by an introduction to SMI. A 5-minute presentation video was also shown to the participants to give an overview of the SMI technology, its functionalities, how it can be used to monitor older adults, and how it can help OTs and PTs to monitor and coach their older adults.

### Data Analysis

Both interviews were audiotaped, transcribed verbatim, and anonymized before analysis. The transcripts were analyzed using thematic analysis [31,32]. We used thematic analysis to understand the barriers to and facilitators of implementing or introducing SMI. The first stage was familiarization with the data, followed by initial coding. Codes were organized in categories (theme identification) and recurring themes (refer to Multimedia Appendix 2 for an overview of themes, categories, and some example quotes). The coding, theme identification, and themes were discussed with MP and MvH (FG 1) and MAC and AQ (FG 2). Discrepancies were resolved until agreement was reached. The final themes were discussed with and agreed upon by the whole research team. The final themes were not shared with the participants owing to feasibility considerations.

## Results

### Focus Group 1

#### Overview

Participants were 9 female OTs, with a median practice experience of 10 (range 1-18) years (Table 1). Before beginning the FG session, participants signed an informed consent form. The participants shared their experiences with the SMI and their reflections and opinions about delivering the intervention. The analysis led to five themes:

1. The transition from inpatient rehabilitation to home rehabilitation
2. Content of the SMI
3. Facilitators of implementing an SMI for rehabilitation
4. Barriers to implementing an SMI for rehabilitation
5. Recommendations for further implementation

**Figure 2:**
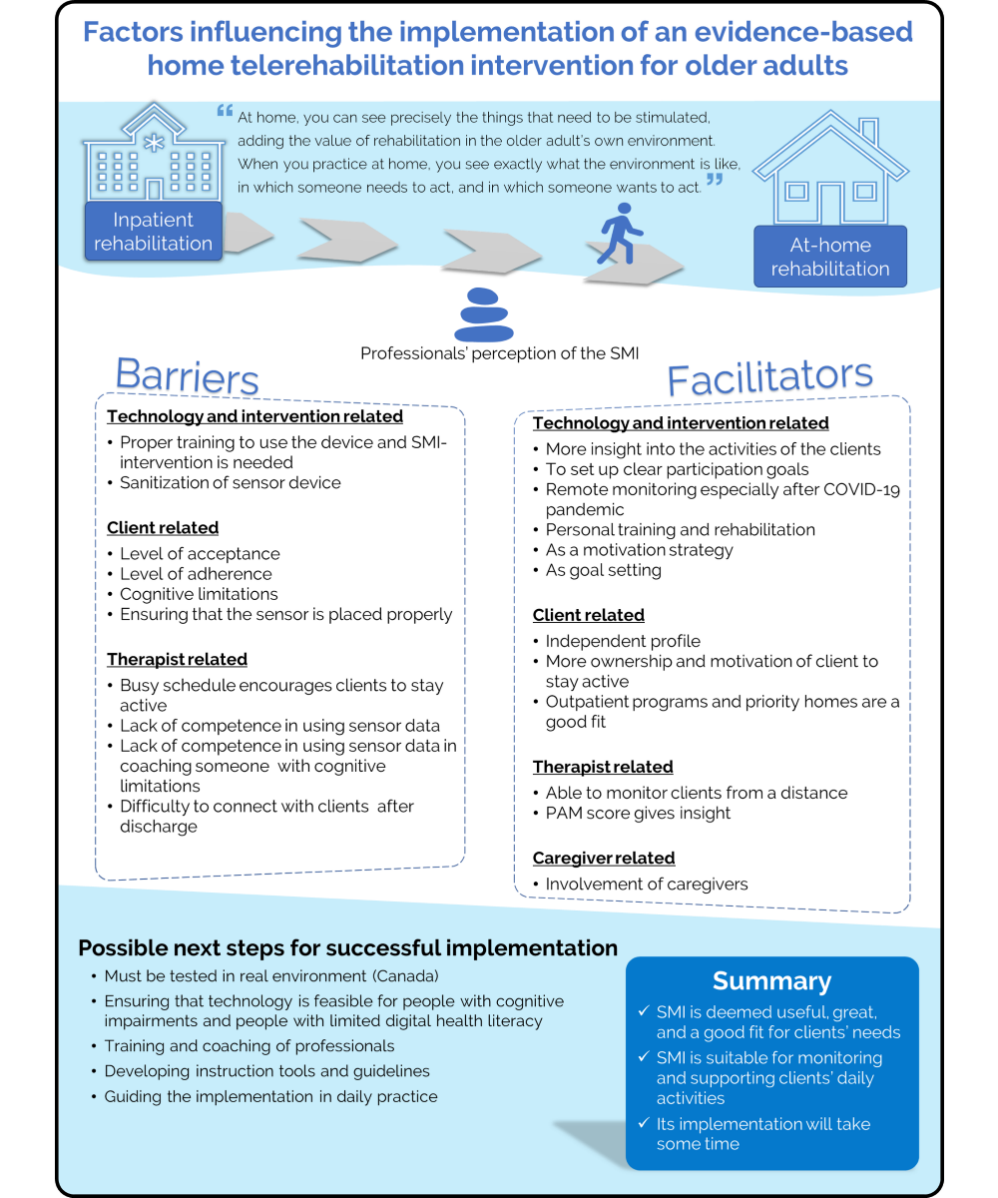
Summary of the results. PAM: physical activity monitor; SMI: sensor monitoring intervention.

Anonymous quotes from participants will be used with the code of the participant. Table 1 shows the codes and background information of the participants of the FGs, and Figure 2 provides a visual summary of the results.

**Table 1:** Characteristics of participants in focus groups 1 and 2.

Focus group and participant ID		Experience (years)	Profession	Type of older adults	Work location
**1**					
	A	8	Occupational therapist	Older adults with orthopedic (trauma and elective), neurological, and complex health problems	Geriatric rehabilitation center A
	B	14	Occupational therapist	Older adults with orthopedic (trauma and elective), neurological, and complex health problems	Geriatric rehabilitation center A
	C	1	Occupational therapist	Older adults with orthopedic (trauma) and neurological problems	Geriatric rehabilitation center B
	D	18	Occupational therapist	Older adults with orthopedic (trauma and elective), neurological, and complex health problems	Geriatric rehabilitation center B
	E	14	Occupational therapist	Older adults with orthopedic (trauma and elective) problems	Geriatric rehabilitation center C
	F	9	Occupational therapist	Older adults with orthopedic (trauma and elective) and complex health problems	Geriatric rehabilitation center A
	G	6	Occupational therapist	Older adults with orthopedic (trauma and elective), neurological, and complex health problems	Geriatric rehabilitation center D
	H	7	Occupational therapist	Older adults with orthopedic (trauma and elective), neurological, and complex health problems	Geriatric rehabilitation center E
	I	12	Occupational therapist	Older adults with orthopedic (trauma and elective), neurological, and complex health problems	Geriatric rehabilitation center F
**2**					
	J	26	Physiotherapist	Older adults with fractures and neurologic conditions	Geriatric rehabilitation
	K	30	Occupational therapist	Geriatric older adults	Geriatric rehabilitation
	L	10	Occupational therapist	Geriatric older adults	Geriatric rehabilitation (previously, acute care and private practice)
	M	22	Occupational therapist	Geriatric older adults	Geriatric rehabilitation
	N	11	Manager of PRIME Care and former clinical service lead for occupational therapy	Geriatric older adults	Deer Lodge Centre

#### Transition From Inpatient Rehabilitation to Home Rehabilitation

The participants generally focused on the added value of at-home rehabilitation, after discharge. They expressed that they were used to only providing inpatient rehabilitation, and by conducting this SMI at home, they experienced the added value of at-home rehabilitation. Participant E said the following:

You can apply some part you practiced in the clinic at home. There [at home] you can see the bottlenecks, precisely the things that need to be stimulated. Then you can apply things much better with someone.

Participant B added the following:

You can practice the task of going to the bathroom in a rehabilitation department that has been made as ideal as possible with a lot of space and adjustments, but it is often different at home.

Participants stated that they became more aware of the added value of treating the clients in their own environment also, for the target group orthopedic rehabilitation. Participant B said the following:

SMI has made me more aware that, I think, with this target group after hip fracture, it is perhaps just as important as with the stroke target group. In the past, we used to see almost no one at home. Still, the idea of continuing the home-based treatment after rehabilitation was more common among people with cognitive problems and stroke older adults because there were more generalization problems. Now, you can see more going on at home than you might have initially expected.

#### Content of the SMI

After focusing on the benefits of at-home rehabilitation in general, participants mentioned different aspects of the content of the SMI specifically.

Most participants indicated that the SMI helped to focus more on future participation goals that focus on what people want to do again in the future, rather than focusing on primary activities of daily living, as illustrated by participant G:

In our regular work, we are much more focused on practical daily functioning, such as getting in and out of bed and going to the toilet. The goals in the SMI are much more oriented towards the future, a few steps further. For example, revisiting family, traveling, or cooking, extensive cooking, it is a different branch of “sport.”

Most participants mentioned that the added value of the intervention helped to get the clients more involved and take more ownership. Participant F stated the following:

The coaching procedure; actively return to those goals each time during the therapy session; where are we now, and how can we take another step forward? I thought that worked well to get someone more involved and in charge of their rehabilitation process.

Participant H noted the following:

I notice a more responsibility on the client’s part and more client control. And I notice that the motivation comes more from the client.

Participants mentioned different aspects of the added value of the sensor monitoring data that provide them additional insight and make it more concrete. Participant I said the following:

I liked the sensor- score. Because without the sensor- score, I sometimes found it quite challenging to shape coaching properly. The moment you made certain things clear with the sensor-score, also for the client, I found it a perfect aid because you could look at it and say: look here, you did almost nothing for two days, and on Wednesday, you suddenly did a lot. What did you do on Wednesday? And how did you make sure you get more done on the other days? The objective data makes it more concrete for me. Just a little more concrete and insightful.

Participant E added the positive value of the sensor data to the therapist as having more upfront information:

You can very well take that sensor information into a conversation. The sensors give additional objective information instead of just a perception. That’s also very important.

Some of the participants explained that the SMI contributed to more involvement, motivation, and taking control of the client, as participant I said the following:

I like it because I had a man who was also cognitively impaired, and who became very enthusiastic about the sensor score and asked: “can I log in at home and keep track of my score?” That motivated him. And not only to start exercising but also to keep himself busy with his rehabilitation. I visited him, and he said: “well, yesterday I had a dip in my graph, because...but the day before I went there and there, I needed to rest.”

The visualization of the sensor data was helpful for therapists to connect to the goals of the client, as participant E illustrated the following:

Well, if someone has a goal, e.g., I want to exercise more, I want to build up my condition, then you can show that to someone, and then you can also say: gosh, I see that you are indeed building up, you have planned a rest day, or you have taken a rest day, well that’s also good for recovery.

Participants experienced the coaching with sensors as providing a deepening in their contact with the clients, as participant G explained the following:

I found it does tighten the contact with someone, where you would otherwise remain more superficial: can you manage to go to the toilet and wash yourself and dress, and maybe it is helpful if there is a shower chair in the shower, you now go more deeply into the conversation with people I think: gosh, what makes it so that you can’t do it now or that you have moved more that day.

#### Barriers to Implementing an SMI for Rehabilitation

The FG discussion identified some barriers to implementing an SMI. The barriers identified were categorized into 2 groups: client-related and therapist-related barriers.

The client-related barriers were (1) the level of vulnerability of the clients, (2) cognitive limitations, and (3) client’s level of acceptance and adherence. Most participants experienced difficulties in conducting the intervention with people with cognitive limitations, as illustrated by participant G*:*

I found it very difficult to coach someone with cognitive limitations. A bit of self-reflection is difficult to stimulate, so realistic goal setting is challenging if one has no insight into his functioning.

Participant B added the following:

Initiative, I think. There are people who at a certain point in time became very passive sitting in a chair and couldn’t think of their own way to do their daily activities and then usually say: “you tell me what I have to do.” Then it becomes challenging to let someone be really active with his rehabilitation and to start thinking about it: how can I do that?

Moreover, participant A mentioned the client’s level of acceptance or adherence:

Sometimes the client does not understand why they are wearing a sensor.

The therapist-related barriers were focused on the competence in using the sensor data in coaching the client. Participant E said the following:

Yes, I did start thinking very consciously about how I use the data. If you indeed see that someone has done a lot one day and very little the next, then you need to know...how I can discuss this with someone without sounding like: why did you do so little that day? Because that is not at all what you want to say.

#### Facilitators of Implementing an SMI for Rehabilitation

Apart from the barriers, some facilitators of implementing an SMI for rehabilitation were identified in the FG discussion. These facilitators were categorized into two groups: (1) client-related and (2) informal care–related facilitators. The client-related facilitators were people who were already interested and motivated and had good cognition. Participant B said the following*:*

Some clients were very interested and motivated in the SMI.

The level of cognitive functioning was mentioned as a facilitator:

The intervention was easy to apply when people had good cognitive functioning.

Participants stated that they see a shift in seeing more vulnerable people who did not function independently before admission. People who were independent before admission found the intervention easy to apply. Participant C said the following:

The intervention was easy to use with people who were independent before admission.

Participants mentioned that the involvement of family or informal caregivers makes SMI easy to use. Participant H said the following:

The intervention was easy to use when family or informal caregivers were involved.

#### Recommendations for Further Implementation

The recommendations for further implementation emanating from the FG discussion were categorized into three groups: (1) organization, (2) involvement of the multidisciplinary team, and (3) training of therapists.

Participant G said the following:

In practice, who is responsible for the technology? How do you arrange that, the technical part, the ICT part? That gives much peace when you have some clarity on that.

Regarding the involvement of the multidisciplinary team, participant H said the following:

In terms of implementation, I also think that you have to take the team with you because, as a multidisciplinary team, you give advice and direction to the process with the client.

Regarding the training, participant G mentioned the following*:*

And I really liked that training of the SMI. I would have liked to see more examples, something with videos or something like that.

Participant E told the following:

And on the follow-up training day, there was also a section on cognitive problems, I found that very useful. I think that should also be included in the basic training because that makes up a large part of this target group.

### Focus Group 2

#### Overview

FG 2 involved 5 participants (n=3, 60% women and n=2, 40% men) with 10 to 30 years of experience in GR, including 4 (80%) OTs and 1 (20%) PT (Table 1), who agreed to participate in the FG study and gave their written consent to participate. They all signed consent forms electronically before gathering for the FG. All the participants (5/5, 100%) had experience in the field of rehabilitation and GR. The thematic analysis enabled patterns (themes and resulting categories) across the data set to be constantly compared and drawn together to describe users’ perceptions and perspectives about the usability of the devices [33]. Primary themes that emerged from the data were categorized into 2 groups: advantages and barriers. These groups were further subdivided as follows.

Overall, 8 final categories or subthemes were identified as advantages of using SMI technology and 7 final categories or subthemes were identified as barriers to using SMI in Manitoba. The advantages of using the SMI technology were categorized into three groups: (1) motivation, (2) other programs, and (3) client monitoring. The barriers to using SMI technology were categorized into 3 groups: devices or materials-related, therapy-related, and personal or client-related barriers.

FG group successfully analyzed the technology and gave valuable feedback regarding the barriers to and facilitators of using SMI technology in Manitoba. They effectively provided information about the current practice and if the new intervention will be successful in this community. The following sections show the advantages of and barriers to using SMI.

#### Advantages of Using SMI

The advantages identified in the FG discussion were categorized into three groups: (1) motivation, (2) fit with existing programs, and (3) monitoring clients.

#### Motivation

The views of our FG participants about the implementation of SMI technology in Manitoba and its barriers and facilitators indicated that this technology has the potential to be useful for older adults in terms of motivation and goal setting, as stated by participant N who “sees this technology as a goal-setting tool” and participant J who mentioned that older adults “could monitor the activity level that’d be beneficial as a motivator.” According to them, this intervention is suitable for the younger population of older adults (aged 65-75 years). They will be able to see and know how active or inactive they are. The intervention was found to be suitable as the OTs and PTs will be able to monitor their clients. OTs and PTs agreed that this intervention would be easy to implement in the younger population of older adults and those who are active in terms of walking and have the habit of staying active. Ensuring that the older adults walk could be a challenge in the absence of a caregiver or supporting family member. Nevertheless, this intervention can be used as a goal-setting approach.

#### Fit With Existing Programs

Some ongoing programs such as “outpatient programs” and “priority homes” can benefit from this technology. The technology was also found to be suitable for personal training and rehabilitation in general. Those outpatient programs that see older adults for extended periods and can invest time in monitoring older adults for long term can benefit from this intervention. Priority home is another type of rehabilitation program in Winnipeg, which offers at-home care, and they can use this technology for GR and monitoring of older adults at home. They continue to monitor older adults for months after discharge. Participant K stated that she “could see it definitely being useful in that type of setting where you are kind of personal training/rehabbing people.”

#### Monitoring Clients

Before the COVID-19 pandemic, people used to stay in rehabilitation centers for months and had time for improvement. However, now, owing to the COVID-19 context, changes have been made, and older adults do not stay in rehabilitation centers for extended periods. The intervention could be helpful in terms of monitoring older adults after discharge. Furthermore, this technology will be suitable for specific populations such as the younger population of older adults and people who live with their family members to support them. Participant M thinks that “it might be worth exploring for the right patient like people, maybe, who are some of our younger geriatrics, maybe more tech-savvy or have a supportive caregiver.” Moreover, it would be essential to know the patient history. Implementing this intervention to monitor older adults can aid in developing the patient’s history over time. OTs and PTs will know whether the person is active or inactive and, then, will be able to work with the person accordingly using the SMI. Using SMI technology, good awareness of a person’s history can help OTs and PTs monitor the clients. The intervention was found to be good for monitoring people by the OTs. Participant M enthusiastically stated the following:

The idea of being able to monitor how much are people doing every day is great; we would love that.

#### Barriers to Using SMI

Apart from the advantages of using SMI technology, some barriers were also identified during the FG discussion. The barriers identified in the FG discussion were categorized into three groups: (1) device or material-related, (2) OT-related, and (3) patient-related barriers.

##### Device or Material-Related Barriers

An important point raised during the discussion was that, given the COVID-19 situation, it would be critical to ensure that the intervention belt remained sanitized, as the older adults would be wearing it daily and performing all their daily activities while wearing the SMI intervention belt. The sensor score states that one will get a PAM score of 6 with half an hour of walking, which means a PAM score of 1 will be for 5 minutes of walking. Ensuring that the person walks for 5 minutes straight to get a PAM score of 1 could be a challenge. A person might be active with intervals, for 2 to 3 minutes, probably going from one room to another, but that might not give the PAM score. Understanding the scores and numbers could be a challenge. Moreover, it could be a challenge for OTs and PTs to ensure that the belt has been placed correctly. Using SMI will not be a challenge for the younger population of older adults, but for the older population and those with cognitive impairment, this could be a challenge; they also need to consider whether the device is missing.

##### OTs and PTs—Related Barriers

According to our participants, OTs and PTs need time to monitor the older adults after discharge and to read graphs of their daily activities while also seeing or monitoring the people who are physically present. This will require extra time, and the schedule of OTs and PTs is usually very busy. In addition, it is difficult to track and stay in contact with the patient on day-to-day basis after the patient is discharged. Ensuring that older adults stay in touch with their OTs in regular basis will be a challenge. Participant K stated the following:

Once the patient is gone from us that bed gets filled with somebody else, and we don’t have any interaction with them. Once they’ve been discharged from Deer Lodge.

Participant K also noted the following:

Days are usually filled, doing a lot of assessments, so, when the beds are full to the day is filled with and you’d have to like things like how long this monitoring would continue.

Another challenge that emerged from our FG discussion is that SMI will be difficult to use for those older adults who live independently with no family member or caregiver. Another challenge that the FG participants stated during the discussion was the challenge with the older adults with cognitive impairment. Participant M stated the following:

It would be difficult to use them with clients with cognitive impairment, or people have no supports, to make sure it’s being done properly, etc. those sorts of things.

##### Personal or Client-Related Barriers

Another challenge that emerged from our FG discussion is that the sensor technology will be difficult to use for those clients who live independently with no family member or caregiver. A substantial challenge that the FG participants stated during the discussion was the challenge with the older adults with cognitive impairment. Participant M stated the following:

It would be difficult to use them with clients with cognitive impairment, or people have no supports, to make sure it’s being done properly etc. those sorts of things.

A general challenge that emerged from our FG was the practicality of SMI. Participant N thinks that “consistency and having sensors put on clients” should be considered. In addition, “not having gone missing” is another challenge according to participant N. Older adults sometimes might forget to wear the belt. In that case, OTs and PTs might be unable to monitor their clients daily. Moreover, the older adults will be discharged and will be at home, not at the rehabilitation centers; thus, it will be a challenge to ensure that those people wear the intervention belt so that the OTs and PTs can monitor them regularly. This is more of a challenge for people with cognitive impairment.

Figure 2 depicts the factors influencing the implementation of the SMI. It summarizes the barriers to and facilitators of implementing SMI for at-home rehabilitation. The figure also suggests a list of possible next steps to be considered to support the implementation of SMI.

## Discussion

### Principal Findings

This study aimed to depict the factors that influence the implementation of an evidence-based home telerehabilitation intervention for older adults (SMI). The information gathered was mapped as barriers to and facilitators of using SMI. We gathered the perspectives of rehabilitation professionals working in two different health ecosystems: (1) rehabilitation professionals in the Netherlands who have previously worked with SMI (FG 1) and (2) rehabilitation professionals in Manitoba (Canada) who attended a 30-minute web-based presentation of SMI before the beginning of the FG but who have not previously worked and did not have experience in working with SMI (FG 2). The qualitative information collected in both contexts provided information about their perceptions of SMI characteristics and the determinants of successful implementation of this telerehabilitation intervention. The information also allowed us to identify the barriers to and facilitators of using SMI.

The participants expressed different characteristics of the telerehabilitation intervention that contributed to making the intervention successful in the Netherlands for the at-home rehabilitation of older adults and potentially successful in Manitoba:

1. The focus of at-home telerehabilitation intervention is on future participation goals rather than focusing on primary activities of daily living.
2. The technology support provides the rehabilitation professionals with objective and additional insight into the daily functioning of the older adults at home, and rehabilitation professionals from both countries find this promising.
3. The technology contributes to more involvement of the person in rehabilitation and can be used as a goal-setting tool underpinning motivation in clients.
4. The coaching, combined with the sensors’ information, deepens their contact with older adults.

According to the rehabilitation professionals, these intervention characteristics facilitated the mechanisms supporting older adults’ recovery at home. This result is consistent with previous studies of the experiences and perspectives of older adults after hip fracture [12]. The interviewed older people positively valued SMI and indicated that the technology served as a strategy to enable independent living. The participants perceived that the system contributed to their sense of safety as an important premise for independent living [12]. Older adults mentioned resources for their recovery, such as coaching, motivation, and technology, that supported them to become more active in developing motivation for engaging more fully in their rehabilitation process [12]. However, different factors influence the implementation. A recent Cochrane review of people after hip fracture [34] recommends to continuously evaluate the effectiveness of the various strategies used for rehabilitating people with hip fractures. They found little to no difference between supported discharge and multidisciplinary home rehabilitation versus usual care for people living in their own homes and no or minimal difference between multidisciplinary rehabilitation versus usual care for nursing home residents. Moreover, a recent systematic review, especially for people in GR [35], concludes that outpatient GR was as effective as usual care and possibly more cost-effective. However, in both reviews, no strategies supported by technology were included.

Digital telerehabilitation solutions such as SMI can allow older adults to get discharged soon from the facility while their therapists will still be able to monitor and coach the older adults from a distance. In the Dutch and Canadian contexts, this study shows the need for—and interest in—using this technology to support older adults in their rehabilitation at home. Rehabilitation professionals stated that they became more aware of the added value of rehabilitating the clients in their environment and using this technology to adapt to the pandemic and postpandemic contexts and demographic trends. Previous researchers also have identified the benefit of telerehabilitation in delivering cost-effective home-based interventions, thus encouraging the transfer and maintenance of the rehabilitation achievements to the home context [36].

As expected, the COVID-19 pandemic emerged as an accelerator for adopting technologies to support remote care, particularly telerehabilitation. Before the COVID-19 pandemic, telerehabilitation had already gained popularity in rehabilitation and occupational therapy services, enabling independence at home through person-centered intervention [35]. Therefore, we are expecting digital technology to take more place to support telerehabilitation. Therefore, in this study, we inquired about the role that SMI would play in at-home telerehabilitation and its acceptance and implementability. Participants in the Netherlands experienced the added value of using SMI to gain more insight into the functioning and participation of the older adult at home. However, the opinions of FG-2 participants about the acceptance of this technology were mixed. Some found it helpful, whereas others liked the opportunity of monitoring the older adults and checking in on them every day. According to FG 2, the technology can be implemented in some running programs, such as the “Outpatient Program” and “Priority Home,” which monitor people in the community after discharge. “Priority Home” is a person-centered collaborative philosophy focused on keeping people—specifically older adults with high needs—safe in their homes for as long as possible with community support [37].

Before the COVID-19 pandemic, in Canada, older adults used to stay for more extended periods at rehabilitation centers and had time for treatment and improvement. Moreover, OTs are used to ensure that the older adult is physically well before leaving the facility. This is similar to the context in the Netherlands. Emerging literature mentions that health care providers turned to technology to stay in touch with older adults [2,38,39]. Older adults were discharged sooner than before to avoid close contact during the COVID-19 pandemic. All the FG participants in Canada perceived monitoring and coaching older adults after being discharged as a valuable idea and a safe practice to standardize beyond the pandemic. They believe it to be an excellent time to have such technology; older adults want to be at home, and if that is possible with the help of portable technology that will connect the older adults to their health care providers, that technology deserves to undergo an implementation trial [40]. In Canada, FG participants stated that while older adults are in the rehabilitation center, they have a team to monitor them, but once the person is discharged, therapists have little consistent interaction with their clients. Therefore, it is beneficial to use telerehabilitation to stay connected after discharge to ensure that people are doing well, as experienced during the COVID-19 pandemic. Following the pandemic, this novel practice should be regarded as a new standard.

The analysis showed facilitators of and barriers to the implementation of the telerehabilitation intervention. These included (1) personal or client-related, (2) therapist-related, and (3) technology-related aspects. All FG participants noted that SMI implementation is feasible if therapists can monitor clients remotely, especially as part of existing programs (eg, outpatient programs) and if the clients are independent and willing to engage in a technology-enabled remote monitoring program. The involvement of a family caregiver has also been noted as a facilitator of implementing SMI. In terms of the intervention, it appears that setting up clear participation goals and a motivational strategy is a key facilitator of remote monitoring. It is well established that motivation promotes better telerehabilitation outcomes, as does patient involvement in decision-making centered on goal setting [41]. Client participation in telerehabilitation is viewed as a facilitator of their adherence to the programs, which determines rehabilitation success. According to the literature, the success of physical rehabilitation programs depends on clients completing the planned therapeutic exercises [42]. SMI provides insight into daily activity, which is thought to promote adherence among independent clients. The results of the RCT were positive for the intervention group [22], and the older adults indicated that the technology served as a strategy to enable independent living [12]. However, both FG participants noted that client’s level of vulnerability, cognitive limitations, and level of acceptance and adherence are the most critical barriers to implementation. Most participants indicated that older adults with cognitive impairment would have difficulty in adapting to technology. However, now and in the future, there will be more people with complex diagnoses, with a mix of cognitive and physical deficits, and more people will stay and return to the community. Therefore, it is important that the technology should be accessible and easy to use by people with limited digital health literacy [43].

Previous studies demonstrated that older adults with limited experience could be taught to use technology successfully [44]. Rehabilitation professionals felt less confident and competent in delivering a telerehabilitation intervention to these older adults. Our findings are consistent with those of other studies that found that some rehabilitation professionals perceived older adults’ lack of confidence in a technology or their old age as a barrier to using that technology and, as a result, may be hesitant to incorporate telerehabilitation into their practice with older adults [45]. These findings must be balanced between the 2 sites in terms of years of clinical experience and technological use. A cross-sectional survey was conducted across Canada to determine Canadian OTs’ knowledge and practice of information and communication technology (ICT) with older adults and factors associated with its recommendation [46]. Of 387 OTs, only 12.4% reported recommending ICT in practice. According to the findings, clinicians with more clinical experience were more likely to recommend ICT. Surprisingly, Canadian participants had more experience than their Dutch counterparts but were more hesitant or reluctant to use the technology with older adults and people with cognitive limitations. The difference between the 2 sites could be owing to environmental differences. According to the same Canadian survey, clinician services, work environments, and client diagnoses were all factors associated with ICT recommendation [46]. The Dutch National Institute for Public Health and the Environment (*Rijksinstituut voor Volksgezondheid en Milieu*) conducted the 2021 eHealth monitor survey to monitor the development and uptake of eHealth across the Netherlands [47]. In comparison with 2019, an increase in the use of eHealth was reported. For example, the use of video calling increased greatly in 2021, and more organizations now have so-called patient portals, apps, or secured websites for their clients. Although the use of eHealth has increased substantially, the COVID-19 pandemic has given it an extra boost. One of the conclusions of the annual report was that clients and care providers are optimistic about this increase, but they also mention some concerns. Clients are often unaware about eHealth options, and rehabilitation professionals found that eHealth creates a heavy workload. Better knowledge and change in working methods are needed to improve this situation [47]. This is consistent with this study, where the FG participants suggested proper training on the use of SMI in their practice [48].

Regarding technology-related barriers, SMI technology does not tell us the number of steps a person walks, but the PAM score on SMI technology indicates how much a person moved per day. The PAM score provides insight into the overall activity and how well a person is progressing. This information helps create achievable goals for speedy recovery. PAM sensor registers the amount of activity and provides insight into the intensity of the activity performed throughout the day. This information was perceived as valuable by the rehabilitation professionals of both FGs. Activity monitors may not accurately detect steps in older adults who walk slowly, as stated in the literature [49], but SMI quantifies the intensity of movement, making it capable of monitoring the activity regardless of the walking speed. According to literature, long walks last longer in hospitals than at home after discharge, whereas short walks are usually more frequent and short at home [50]. It was mentioned by the FG participants that the therapists would want the older adults to be active after getting discharged, but once they leave the facility, they do not do that often. Overall, all the FG participants believed that telerehabilitation could be helpful for older adults. It will help OTs and PTs to have more insight into the daily physical activities of the individuals after getting discharged and allow older adults to be treated in their own homes.

Making telerehabilitation beneficial, functional, and feasible for people with cognitive impairment could be the next important step in making the telerehabilitation technology better and more suitable for such a population. We should further develop the graphs of the sensor technology to give better information to therapists to help them understand what the sensor scores mean and how the scores are situated versus the rehabilitation goals already set up with the older adult. Training on how to use the telerehabilitation intervention with people with cognitive impairment and implementing the intervention in their daily practice is needed. Therefore, we must develop instructional tools and guidelines with the rehabilitation professionals and older adults to ensure implementation in their working routines. Collaboration among all stakeholders in further developing the telerehabilitation intervention is essential for its implementation [29]. Our results indicate that the adoption of telerehabilitation technology may take time. It will be good to implement this technology not only for the younger population of older adults (aged 65-75 years) but also to make it feasible for all different groups of the older population, including those with low digital health literacy. Although there will be barriers with some specific populations, such as older adults with cognitive impairment, the technology can be implemented successfully in practice with the proper approaches.

### Strengths and Limitations

One of the strengths of our study was the generation of a valuable understanding of rehabilitation professionals’ experiences and perspectives about implementing a telerehabilitation intervention and the factors contributing to its implementation. Another strength is that we included data from 2 different international contexts, before and after the COVID-19 pandemic, and collected data from participants who had hands-on experience with an SMI and from participants who had not. Internationally, GR is offered as different services; therefore, we can only make general recommendations for implementing an SMI, as obtained from the FG discussions. It is necessary to test the SMI in a specific context. The 2 studies did not include the perspectives of older people, their family members, or decision makers about the SMI technology. However, we investigated the perspectives of older adults in the Netherlands previously [12]. The studies concentrated on rehabilitation professionals because they are involved in both the individual (care delivery to older adults) and system levels. However, more studies are needed to understand the factors influencing SMI implementation from organizational perspectives (eg, policy makers and decision makers) and perspectives of older adults and their family members in Canada.

### Conclusions

Rehabilitation professionals believed that telerehabilitation could be suitable for monitoring and supporting older adults’ rehabilitation at home. The analysis showed facilitators of and barriers to the implementation of the telerehabilitation intervention. These included (1) personal or client-related, (2) therapist-related, and (3) technology-related aspects. To better guide the implementation of telerehabilitation in the daily practice of rehabilitation professionals, the following steps are needed: (1) ensuring that technology is feasible for a population with limited digital health literacy or cognitive impairments, (2) developing instruction tools and guidelines, and (3) training and coaching of rehabilitation professionals.

## Appendix

Multimedia Appendix 1 Topic guide for focus group for exploring occupational therapists’ experience with and opinions about delivering the sensor monitoring intervention for rehabilitation after hip fracture (SO-HIP).

Multimedia Appendix 2 Overview of themes, categories, and quotes from the focus group interviews.

## Abbreviations

COREQ: Consolidated Criteria for Reporting Qualitative Research
FG: focus group
GR: geriatric rehabilitation
ICT: information and communication technology
OT: occupational therapist
PAM: physical activity monitor
PT: physical therapist
RCT: randomized controlled trial
SMI: sensor monitoring intervention

## Data Availability

The data sets generated and analyzed during this study are available from the corresponding author upon reasonable request.
