# Supplementary material for: Perspectives of Rehabilitation Professionals on Implementing a Validated Home Telerehabilitation Intervention for Older Adults in Geriatric Rehabilitation: Multisite Focus Group Study
Source: JMIR Rehabil Assist Technol. 2023 Jul 18;10:e44498. doi: 10.2196/44498 (PMC10394599; doi:10.2196/44498)
Supplement: Multimedia Appendix 1 [file rehab_v10i1e44498_app1.docx]

Appendix 1. Topic guide focus group for exploring occupational therapists’ experience with and opinions about delivering the SO-HIP intervention

| Topic | Open Questions and probing questions | Working Style |
| --- | --- | --- |
| Characteristics of a successful SO-HIP intervention | You all gained some experience with the SO-HIP intervention. Before we discuss the details, here is a first general question:  What are in your opinion the characteristics of a successful SO-HIP intervention?  -What type of results should be achieved? | Brainstorming session and  discussion |
| Which factors or characteristics facilitated the SO-HIP intervention? | What factors contributed to make the SO-HIP intervention successful?   - E.g. factors related to different aspects of the SO-HIP intervention - Factors related to the patient - Factors related to the therapist - Contextual or organizational aspects | Interview, Brainstorming session and  discussion |
| Which factors or characteristics hindered the SO-HIP intervention? | What factors or aspects hindered the SO-HIP intervention?   - E.g. factors related to different aspects of the SO-HIP intervention - Factors related to the patient - Factors related to the therapist   Contextual or organizational aspects | Interview, Brainstorming session and  discussion |
| Experiences with the work of the SO-HIP intervention | - What did you like and what did you dislike about applying the SO-HIP intervention? What went well and what could be improved?  - Experience with coaching: Did you feel the coaching intervention made any difference to the way patients engaged and participated in their rehabilitation? If so, how? If not why?  -Experience with the coaching sessions at home: How did you experience the extra coaching sessions at home? Did you use these extra sessions, and if so, did it make any differences in your opinion?  -How did you experience the extra telephone sessions? Did you use them and did it make any difference in your opinion?  -Experience with the sensor technology: How did you experience the coaching with the sensor technology? Did it make any difference in your opinion? Did you experience that the coaching based on the sensor data made any difference to the way patients participated in their rehabilitation?  -Experiences with cognitive impairment: How did you experience the SO-HIP intervention with patients with cognitive impairments? How did the intervention go? | Interview and discussion |
|  | Which changes would contribute to the implementation of the SO-HIP intervention? | Interview |
|  | Other things, that you think will be important in working with this SO-HIP intervention? | Interview |
