# Supplementary material for: Perspectives of Rehabilitation Professionals on Implementing a Validated Home Telerehabilitation Intervention for Older Adults in Geriatric Rehabilitation: Multisite Focus Group Study
Source: JMIR Rehabil Assist Technol. 2023 Jul 18;10:e44498. doi: 10.2196/44498 (PMC10394599; doi:10.2196/44498)
Supplement: Multimedia Appendix 2 [file rehab_v10i1e44498_app2.docx]

Appendix 2: Overview of themes, categories, and quotes from the focus group interview

| Theme | category | codes | Some illustrative quotations therapists |
| --- | --- | --- | --- |
| 1. Transition from inpatient rehabilitation to home rehabilitation | Added value of home rehabilitation following inpatient rehabilitation | Practice at home  Rehabilitation in patient’s own environment  Back to living at home as a goal  Awareness of the added value of home rehabilitation  Practicing at home is more effective | R5 “You can apply some part you practiced in the clinic at home. There (home) you can see the bottlenecks, precisely the things that need to be stimulated. Then you can apply things much better with someone”.  R5“And, when you practice at home, you see exactly what the environment is like, in which someone needs to act, and in which someone wants to act.”  R4“It's much more the natural performing you're doing in the home situation. Yes, the goals also come to live more at home.”  R5 “The SO-HIP intervention did help us; actually, we knew it already, but to become even more aware of the added value of treating them (the clients) in their environment. It did change things for us. Yes, previously, we used to see very few people at home.”  R2“SO-HIP has made me more aware that, I think, with this target group after hip fracture, it is perhaps just as important as with the stroke target group’. In the past, we used to see almost no one at home. Still, the idea of continuing the outpatient treatment after rehabilitation was more common among people with cognitive problems and stroke patients because there were more generalization problems.  Now, you can see that there is more going on at home than you might have initially expected. Somehow you know that, but now you become more aware of it’.  R2“You can practice the task of going to the bathroom in a rehabilitation department that has been made as ideal as possible with a lot of space and adjustments, but it is often different at home.  R4For example, a kitchen with us is quite spacious, but in the home situation, it can be tiny, and then it is in a different way that you have to put your rollator walker down or turn it if you want to make it safe. So, then it is more effective to practice that at home indeed'. |
| 2. Content of the sensor monitoring intervention | The added value of the sensor monitoring intervention | Assessing fall risk is much more realistic in the home situation | R2“Sometimes, it turns out that everything is going very well in the clinic. Everyone is very enthusiastic that the person can go home, and then you indeed go on a home visit or look at the home, and then it turns out that the situation in the home is different from what is expected, and you still see things that can cause a risk of falling”. |
|  |  | More motivation to practice at home  Focus on meaningful goals; the intervention goals aim at what people want to do again in the future.  Added value of the coaching intervention helped to get someone more involved  More ownership by the client  Plus value of the sensor; provides additional insight and makes it concrete  The sensor contributes to involvement and motivation, taking control  Plus value of sensor data for the therapist; as a therapist, you have more upfront information  Make it visual and connect with the sensor data to the goals that someone has.  Coaching with sensors provides a deepening in our contact | R4“People sometimes say, 'oh, when I am home, I can practice, but at home, it's different. And when they are at home, they are like ‘gosh, this is what I do most of the time.”.  R7“In our regular work, we are much more focused on practical daily functioning, such as getting in and out of bed and going to the toilet. The goals in the sensor monitoring intervention are much more oriented towards the future, a few steps further. For example, revisiting family, travelling or cooking, extensive cooking, it is a different branch of “sport”.  R6“The coaching procedure; actively return to those goals each time during the therapy session; where are we now, and how can we take another step forward? I thought that worked well to get someone more involved and in charge of their rehabilitation process.”.  R8“I notice a more responsibility on the client's part and more client control. And I notice that the motivation comes more from the client”.  R9“I liked the PAM score. Because without the PAM score, I sometimes found it quite challenging to shape coaching properly. The moment you made certain things clear with the PAM score, also for the client, I found it a perfect aid because you could look at it and say: look here, you did almost nothing for two days, and on Wednesday, you suddenly did a lot. What did you do on Wednesday? And how did you make sure you get more done on the other days? The objective data makes it more concrete for me and also for the clients. Just a little more concrete and insightful.”  R1“Yes, you have visuals; it's not just the verbal”  R9I like it because I had a man who was also cognitively impaired, who had first been delirious after his operation, and who became very enthusiastic about the PAM score and asked: ‘can I log in at home and keep track of my score’? That motivated him. And not only to start exercising but also to keep himself busy with his rehabilitation. I visited him, and he said: ‘well, yesterday I had a dip in my graph, because...... but the day before I went there and there, I needed to rest’. That I thought: you are keeping yourself busy with your rehabilitation.  R5You can very well take that sensor information into a conversation.  The sensors give additional objective information instead of just a perception. That's also very important  R5“Well, if someone has a goal, e.g., I want to exercise more, I want to build up my condition, then you can show that to someone, and then you can also say: gosh, I see that you are indeed building up, you have planned a rest day, or you have taken a rest day, well that's also good for recovery. Then you can pick it up that way. Even though someone can be tired for a day, it doesn't have to be a very active day physically”.  R7“I found it does tighten the contact with someone, where you would otherwise remain more superficial: can you manage to go to the toilet and wash yourself and dress, and maybe it is helpful if there is a shower chair in the shower, you now go more deeply into the conversation with people I think: gosh, what makes it so that you can't do it now or that you have moved more that day”. |
| 3. Barriers to implementing a sensor monitoring intervention for rehabilitation | Barriers to the use of the sensor monitoring intervention | Level of vulnerability.  Cognitive limitations  Client’s level of acceptance/adherence  People who do not take the initiative.  Therapists' level of competence in using the sensor data in coaching the person | R2“I see much more vulnerable people with a limited social network; mobility was not all that great, they could manage at home, and then they felll and came to us for rehabilitation”.  R7“I found it very difficult to coach someone with cognitive limitations.  A bit of self-reflection is difficult to stimulate, so realistic goal setting is challenging if one has no insight into his functioning”.  R1“Sometimes the client does not understand why they are wearing a sensor”.  R2“Initiative, I think. There are people who at a certain point in time became very passive sitting in a chair and could't think of their own way to do their daily activities and then usually say: “you tell me what I have to do”. Then it becomes challenging to let someone be really active with his rehabilitation and to start thinking about it: how can I do that”?  R5“Yes, I did start thinking very consciously about how I use the data. If you indeed see that someone has done a lot one day and very little the next, then you need to know... how I can discuss this with someone without sounding like: why did you do so little that day? Because that is not at all what you want to say”. |
| 4. Facilitators of implementing a sensor monitoring intervention for rehabilitation | Ease of use of the sensor monitoring intervention | People who are already motivated  People who were independent before admission  Involvement of family and informal caregivers  Good cognitive functioning | Writing Notes of the therapists  R2“Some clients were very interested and motivated in the so-hip intervention”.  R3“The intervention was easy to use with people who were independent before admission”.  R8“The intervention was easy to use when family or informal caregivers were involved”.  R2“The intervention was easy to apply when people had good cognitive functioning”. |
| 5. Recommendations for further implementation |  | Make someone responsible for the technology  Involve the multidisciplinary team  Include handling cognitive problems in the basic training | R7“In practice, who is responsible for the technology? How do you arrange that, the technical part, the ICT part? That gives much peace when you have some clarity on that”.  R8“In terms of implementation, I also think that you have to take the team with you because, as a multidisciplinary team, you give advice and direction to the process with the client.”  R7“And I really liked that training of the sensor monitoring intervention. I would have liked to see more examples, something with videos or something like that”.  R5 And on the follow-up training day, there was also a section on cognitive problems, I found that very useful. I think that should also be included in the basic training, because that makes up a large part of this target group”. |
